# *RNF152* promoter methylation and gene expression in oral premalignant lesions and oral squamous cell carcinoma: implications for epigenetic biomarker development

**DOI:** 10.3389/fonc.2026.1943424

**Published:** 2026-09-24

**Authors:** Madona Gjona, Seval Turna, Cömert Şen, Ahmet Özkan, Kağan Avcı, Murat Ulusan, Gülsüm Ak, Vakur Olgaç, Semra Demokan

**Affiliations:** 1 Institute of Graduate Studies in Health Sciences, Istanbul University, Istanbul, Türkiye; 2 School of Health Services Vocational College, Pathology Laboratory Techniques Program, Bezmialem Vakif University, Istanbul, Türkiye; 3 Department of Otorhinolaryngology-Head and Neck Surgery, Istanbul Faculty of Medicine, Istanbul University, Istanbul, Türkiye; 4 Department of Oral and Maxillofacial Surgery, Faculty of Dentistry, Istanbul University, Istanbul, Türkiye; 5 Department of Oral Pathology, Faculty of Dentistry, Istanbul University, Istanbul, Türkiye; 6 Department of Basic Oncology, Oncology Institute, Istanbul University, Istanbul, Türkiye

**Keywords:** DNA methylation, epigenetic biomarkers, gene expression, liquid biopsy, oral premalignant lesions, oral squamous cell carcinoma, *RNF152*

## Abstract

**Purpose:**

Aberrant promoter DNA methylation represents an early molecular alteration in oral carcinogenesis and has emerged as a potential source of molecular biomarkers for oral cancer. Although *RNF152* has been implicated in cancer-related molecular dysregulation, its promoter methylation and expression status have not previously been specifically investigated in oral premalignant lesions (OPML) or oral squamous cell carcinoma (OSCC).

**Experimental design:**

*RNF152* promoter methylation and gene expression were evaluated in paired tumor/lesion and matched adjacent normal tissues from 30 patients with OSCC and 30 patients with OPML, together with oral mucosal tissues from 15 healthy controls. Matched saliva and serum samples from the patient cohorts were also analyzed to evaluate *RNF152* methylation as a potential liquid biopsy marker. Quantitative methylation-specific PCR (QMSP) and quantitative real-time PCR (QRT-PCR) were performed, and associations with clinicopathological characteristics, diagnostic performance, and the relationship between promoter methylation and gene expression were investigated.

**Results:**

*RNF152* promoter methylation was detected in 36.7% of OSCC tumors and 70.0% of OPML lesions, whereas no promoter methylation was identified in oral mucosal tissues from healthy controls. Promoter methylation was also detected in matched saliva and serum samples from both patient groups. Reduced *RNF152* expression was observed in 33.3% of OSCC and 30.0% of OPML tissues. Methylation-associated *RNF152* expression loss was identified in 13.3% of OSCC and 30.0% of OPML patients. Among methylation-positive cases, concomitant *RNF152* expression loss was observed in 36.4% of patients with OSCC and 42.9% of patients with OPML. Continuous promoter methylation levels were not significantly correlated with tissue gene expression in either cohort. *RNF152* promoter methylation demonstrated good discriminatory performance for distinguishing OPML from healthy controls (AUC = 0.867, p < 0.001), whereas its discriminatory performance for OSCC was statistically significant but more modest (AUC = 0.683, p = 0.047).

**Conclusions:**

This study provides the first comprehensive evaluation of *RNF152* promoter methylation and gene expression in oral premalignant lesions and oral squamous cell carcinoma. *RNF152* promoter methylation showed greater diagnostic potential for OPML than for OSCC, whereas its performance in liquid biopsy specimens was limited. The incomplete concordance between promoter methylation and gene expression suggests that promoter methylation may contribute to *RNF152* downregulation in a subset of cases but is not the sole determinant of its expression. These findings support further evaluation of *RNF152* as a candidate component of multimarker epigenetic panels rather than as a standalone diagnostic biomarker. Validation in larger, independent cohorts is required to determine its clinical utility.

## 1 Introduction

Head and neck cancer (HNC) represents a major global health burden, accounting for approximately 5% of all cancer cases worldwide (1). Within this disease spectrum, OSCC is among the most prevalent malignancies and represents a particularly important health burden in South Asia, where tobacco and areca-nut/betel-quid chewing constitute major etiological exposures (2). According to GLOBOCAN 2020, Asia accounts for approximately 65.8% of all newly diagnosed oral cancer cases, followed by Europe (17.3%), North America (7.3%), Latin America and the Caribbean (4.7%), Africa (3.8%), and Oceania (1%) (3). Despite considerable advances in surgery, radiotherapy, chemotherapy and targeted therapies, the overall five-year survival rate for OSCC remains only 50%-60%, largely because many patients are diagnosed at advanced stages of disease (4, 5). Consequently, the identification of reliable biomarkers capable of detecting early-stage disease or improving molecular risk stratification remains an important clinical challenge.

OSCC commonly develops through a multistep process involving the progressive accumulation of molecular alterations associated with carcinogenic exposures, including tobacco, alcohol, human papillomavirus (HPV), and betel nut (6). This process may involve oral potentially malignant disorders, collectively referred to in the present study as oral premalignant lesions (OPML). These lesions comprise a heterogeneous group of disorders, including oral leukoplakia (OL), oral lichen planus (OLP) and oral lichenoid dysplasia (OLD), which differ in their histopathological characteristics and risks of malignant transformation (7, 8). Genetic and epigenetic alterations contribute to the molecular evaluation of OPML and the development of invasive OSCC (9). Therefore, identifying molecular alterations associated with oral carcinogenesis remains important for improving early detection and molecular characterization.

Both genetic and epigenetic alterations contribute to the development and progression of oral carcinogenesis (10). Among the various epigenetic mechanisms implicated in cancer, aberrant DNA methylation represents one of the earliest and most extensively characterized molecular events (11). This process involves the addition of a methyl group to the fifth carbon position of the cytosine base, thereby regulating gene expression through alterations in chromatin structure and DNA-protein interactions (12). In cancer, aberrant DNA methylation, particularly promoter CpG island hypermethylation, is one of the best-characterized epigenetic alterations and frequently leads to transcriptional silencing of tumor suppressor genes (13). Consequently, epigenetic dysregulation contributes to tumorigenesis by disrupting multiple cellular processes, including cell cycle regulation, DNA repair, apoptosis, cell adhesion, signal transduction, and cellular differentiation (14). Owing to its relative stability, tissue specificity and detectability in both tissue and body fluids, promoter DNA methylation has emerged as an attractive source of diagnostic and prognostic biomarkers for oral cancer.

Genome-wide methylation studies have identified numerous loci exhibiting altered methylation patterns during head and neck carcinogenesis. Poage et al. (15) demonstrated that global DNA hypomethylation may coexist with locus-specific methylation alterations, highlighting the complex epigenetic landscape of HNSCC. Although *RNF152* was not a primary target of that study, an *RNF152*-associated CpG locus (cg19423014) was identified in their genome-wide methylation dataset, which is publicly available in the NCBI Gene Expression Omnibus (GEO) under accession number GSE25093, among loci significantly associated with LINE-1 methylation (Spearman's ρ = 0.029; FDR q = 0.0042) (15). However, *RNF152* was not specifically investigated or independently validated as a promoter methylation target, and its methylation prevalence and biological or clinical relevance in oral carcinogenesis remained unknown. These observations provided the rationale for selecting *RNF152* for targeted investigation in the present study, in which we evaluated its promoter methylation and gene expression in clinically characterized OPML and OSCC cohorts.

*RNF152* belongs to the RING finger protein family, whose members function predominantly as E3 ubiquitin ligases involved in ubiquitin-mediated protein degradation and intracellular signaling pathways (16, 17). More than 300 human proteins containing RING-type zinc finger domains have been identified, participating in diverse biological processes including protein turnover, DNA repair, transcriptional regulation, and signal transduction. *RNF152* has also been implicated in regulating lysosome-associated signaling pathways and cellular homeostasis (16). Altered *RNF152* expression has been reported in several human malignancies, including lung adenocarcinoma and colorectal cancer, indicating heterogeneous expression and biological roles across tumor types (18, 19). In addition, mutations affecting *RNF152* have been associated with neurological disorders and various malignancies (20). Despite these observations, the precise physiological functions of *RNF152* and the mechanisms through which its dysregulation contributes to tumor development remain incompletely understood. Given the absence of targeted studies investigating *RNF152* promoter methylation and expression in oral carcinogenesis, the present study aimed to comprehensively evaluate *RNF152* promoter methylation and gene expression in clinically characterized OPML and OSCC cohorts and to investigate these molecular alterations. Matched tissue, saliva and serum specimens were further analyzed to assess the detectability and diagnostic potential of *RNF152* promoter methylation in liquid biopsy samples. To our knowledge, this is the first targeted clinical study to characterize *RNF152* promoter methylation across both oral premalignant and malignant lesions while integrating methylation status with gene expression and matched liquid-biopsy analyses.

## 2 Materials and methods

### 2.1 Sample collection

This study included 30 patients with OSCC, 30 patients with OPML and 15 healthy controls. Patients with OSCC underwent primary surgical treatment at the Department of Otorhinolaryngology-Head and Neck Surgery, Istanbul University, Faculty of Medicine, whereas patients with OPML were recruited from Department of Oral and Maxillofacial Surgery, Istanbul University, Faculty of Dentistry. Healthy controls were individuals without a history of premalignant or malignant oral lesions who underwent uvulopalatopharyngoplasty (UPPP) for non-malignant indications at the Department of Otolaryngology, Istanbul University Faculty of Medicine. The collected mucosal specimens were histopathologically confirmed to be normal before inclusion in the molecular analyses. UPPP-derived histologically normal mucosa has been widely used as a non-neoplastic squamous epithelial reference in molecular studies of head and neck squamous cell carcinoma (21–24). This approach permits the acquisition of histologically normal squamous mucosa without subjecting healthy individuals to an additional research-related biopsy. None of the patients with OSCC had received chemotherapy or radiotherapy before surgery, and primary surgical resection was the initial treatment for all patients with OSCC.

Given the exploratory nature of the study and the lack of prior clinical data on *RNF152* promoter methylation frequencies in OPML and OSCC, a reliable gene-specific effect size for conventional *a priori* sample-size estimation was not available. Accordingly, the study cohort comprised all eligible participants recruited during the predefined study period, including 30 patients with OSCC, 30 patients with OPML, and 15 healthy controls. To evaluate the statistical adequacy of the available sample size, a sensitivity analysis was subsequently performed using G*Power version 3.1 (Heinrich Heine University Düsseldorf, Germany) (25). At a significance level (α) of 0.05 and a statistical power (1-β) of 80%, the final cohort of 75 individuals was sufficient to detect an overall effect size of approximately Cohen’s w = 0.36 for differences in *RNF152* methylation frequencies among the three study groups.

The study protocol was approved by the Ethics Committee of Istanbul Faculty of Medicine (Approval No. 2020/640), and written informed consent was obtained from all participants before sample collection in accordance with the Declaration of Helsinki.

Tissue samples consisted of primary tumor tissues and matched adjacent normal tissues from patients with OSCC, lesion tissues and matched adjacent normal tissues from patients with OPML, and normal oral mucosal tissues from healthy controls. Tumor specimens were reviewed by an experienced pathologist, and only samples containing at least 75% tumor cells were included in the study. Peripheral blood and unstimulated whole saliva samples were collected before surgery or biopsy, as applicable. For saliva collection, participants were instructed to refrain from eating or drinking for at least 1 hour before sampling. After rinsing their mouths with drinking water for 1 minute, unstimulated whole saliva was allowed to accumulate for approximately 5 minutes while gently circulating throughout the oral cavity until approximately 5 mL had been collected. Saliva samples were diluted 1:1 with isotonic saline, centrifuged at 1950 rpm for 5 minutes, and the supernatant was discarded. The resulting cell pellet was stored for subsequent molecular analyses. Peripheral blood samples (8–10 mL) were collected into serum separation tubes and centrifuged at 1950 rpm for 30 minutes. The separated serum was aliquoted into 1-mL cryotubes. All tissue, serum and saliva samples were stored at -80 °C until DNA and RNA isolation.

### 2.2 RNA extraction and cDNA synthesis

Total RNA was isolated from lesion tissues, matched adjacent normal tissues, serum and saliva samples obtained from 30 patients with OPML; tumor tissues, matched adjacent normal tissues, serum and saliva samples obtained from 30 patients with OSCC; and oral mucosal tissues, serum and saliva samples obtained from 15 healthy controls using the Norgen Total RNA Purification Kit (Norgen Biotek Corp., Ontario, Canada) according to the manufacturer’s instructions. RNA concentration and purity were assessed spectrophotometrically using a NanoDrop 2000 spectrophotometer (Thermo Fisher Scientific, Wilmington, DE, USA). The A260/A280 and A260/A230 absorbance ratios were used to assess protein and chemical contamination, respectively. RNA samples with A260/A280 ratios of approximately 2.0 and A260/A230 ratios between 2.0 and 2.2 were considered to have acceptable spectrophotometric purity. RNA integrity of tissue-derived RNA samples was additionally assessed by agarose gel electrophoresis before cDNA synthesis. Complementary DNA (cDNA) was synthesized from 1 μg of total RNA using the SensiFAST cDNA Synthesis Kit (Bioline Meridian Bioscience, London, UK), according to the manufacturer’s instructions. The synthesized cDNA samples were stored at -80 °C until QRT-PCR analysis.

### 2.3 DNA extraction and bisulfite treatment

Genomic DNA was extracted from lesion tissues, matched adjacent normal tissues, serum and saliva samples obtained from 30 patients with OPML, tumor tissues, matched adjacent normal tissues, serum and saliva samples obtained from 30 patients with OSCC, and oral mucosal tissues obtained from 15 healthy controls using the conventional phenol-chloroform extraction method following proteinase K digestion. DNA concentration and purity were assessed using a NanoDrop 2000 spectrophotometer (Thermo Fisher Scientific, Wilmington, DE, USA). DNA samples with A260/A280 ratios of approximately 1.8 and A260/A230 ratios between 2.0 and 2.2 were considered to have acceptable spectrophotometric purity. DNA integrity of tissue-derived samples was additionally assessed by agarose gel electrophoresis before bisulfite conversion.

For DNA methylation analysis, 200 ng of genomic DNA from each sample was subjected to bisulfite conversion using the EZ DNA Methylation-Gold Kit (Zymo Research, Irvine, CA, USA) according to the manufacturer’s instructions. Bisulfite-converted DNA was stored at -20 °C until QMSP analysis.

### 2.4 *RNF152* gene expression analysis by quantitative real-time PCR

QRT-PCR was performed to evaluate *RNF152* mRNA expression in lesion tissues, matched adjacent normal tissues, serum and saliva samples obtained from 30 patients with OPML, tumor tissues, matched adjacent normal tissues, serum and saliva samples obtained from 30 patients with OSCC, and oral mucosal tissues, serum and saliva samples obtained from 15 healthy controls. Primers for *RNF152* gene expression analysis were designed using Primer3Plus (https://www.primer3plus.com/), whereas primer sequences for the endogenous reference gene *ACTB* were adopted from a previously published study by Demokan et al. (21). The primer sequences used in this study are provided in **Table 1**.

**Table 1:** Primer and probe sequences used for QRT-PCR and QMSP analyses of *RNF152* and *ACTB*.

Gene	Assay	Oligonucleotide	Sequence (5’ → 3’)
*RNF152*	QRT-PCR	Forward	TCAGCCTCTTGCCTCCTAAAC
*RNF152*	QRT-PCR	Reverse	AATGGCAAGCTCACAGGCAT
*ACTB*	QRT-PCR	Forward	GTCTTCCCCTCCATCGTG
*ACTB*	QRT-PCR	Reverse	AGGGTGAGGATGCCTCTCTT
*RNF152*	QMSP	Forward	AGCGTTCGTTTCGTGGTTTT
*RNF152*	QMSP	Reverse	TCACTAACCTTTCTCCGCGAC
*RNF152*	QMSP	Probe	TCGTCGGTTCGGGGCCGAGTTTGGTT
*ACTB*	QMSP	Forward	TGGTGATGGAGGAGGTTTAGTAAGT
*ACTB*	QMSP	Reverse	AACCAATAAAACCTACTCCTCCCTTAA
*ACTB*	QMSP	Probe	ACCACCACCCAACACACAATAACAAACACA

Before gene expression analyses, QRT-PCR conditions were optimized for *RNF152* and *ACTB*, including primer concentration, cDNA input and annealing conditions. PCR amplification was performed using the LightCycler 480 System (Roche Diagnostics GmbH, Mannheim, Germany) with SYBR Green I Master Mix (Roche Diagnostics GmbH). Cycling conditions consisted of an initial denaturation step at 95 °C for 5 min, followed by 45 cycles of denaturation at 95 °C for 15 s and annealing/extension at 60 °C for 1 min, followed by melting at 95 °C for 10 s, cooling at 55 °C for 20 s, and continuous melting-curve acquisition. Amplification specificity was confirmed by melting-curve analysis. All reactions were performed in duplicate or triplicate.

Fluorescence data were analyzed using the Basic Relative Quantification module of the LightCycler 480 software (Roche Diagnostics GmbH). *ACTB* was used as the endogenous reference gene for normalization. ΔCt values were calculated as Ct(*RNF152*)-Ct(*ACTB*). For paired tissue analyses, ΔΔCt values were calculated as ΔCt(tumor/lesion)-ΔCt(matched adjacent normal tissue), and relative *RNF152* expression was determined using the 2-^ΔΔCt^ method (26). Samples with fold-change values ≥2 were classified as showing increased *RNF152* expression, whereas those with fold-change values ≤0.5 were classified as showing decreased expression. Samples with fold-change values >0.5 and <2 were considered to show no marked change in expression.

### 2.5 Quantitative methylation-specific PCR

QMSP was performed to evaluate *RNF152* promoter methylation in lesion tissues, matched adjacent normal tissues, serum and saliva samples obtained from 30 patients with OPML, in tumor tissues, matched adjacent normal tissues, serum and saliva samples obtained from 30 patients with OSCC, and in oral mucosal tissues from 15 healthy controls.

The promoter region and CpG island of the *RNF152* gene were identified using the University of California, Santa Cruz (UCSC) Genome Browser (http://genome.ucsc.edu/). *RNF152* methylation-specific primers and probe were designed using MethPrimer (27) and Primer3Plus, whereas *ACTB* methylation primers and probe used as the internal reference were adopted from Carvalho et al. (28). The primer and probe sequences used in this study are listed in **Table 1**.

Each QMSP reaction was performed in a final volume of 20 μL containing 600 nM of each primer, 200 nM probe, 10 μL of 2 x SensiFAST Probe No-ROX Mix (Bioline, UK), PCR-grade dH_2_O, and 5 μL of bisulfite-converted DNA. PCR amplification was carried out using the LightCycler^®^ 480 System (Roche Diagnostics GmbH, Mannheim, Germany) with an initial denaturation at 95 °C for 30 s, followed by 45 cycles of denaturation at 95 °C for 15 s, annealing at 64 °C for 1 min, and extension at 72 °C for 15 s, with a final cooling step at 40 °C for 30 s. Fluorescence data were analyzed using the Absolute Quantification/Fit Points module of the LightCycler^®^ 480 software (Roche Diagnostics GmbH, Mannheim, Germany). Each reaction was performed in duplicate or triplicate. Genomic DNA isolated from lymphocytes of a healthy individual was methylated *in vitro* using excess SssI methyltransferase and S-adenosylmethionine (Zymo Research, CA, USA) to generate fully methylated DNA, which was subsequently subjected to bisulfite modification and used as the positive control standard. Serial dilutions (1:10-1:10,000) of the bisulfite-modified fully methylated DNA were used to generate a standard curve for each experiment. Unmethylated lymphocyte-derived DNA was included as a negative control, and no-template controls were included in each experiment to monitor nonspecific amplification and contamination. Assay performance was monitored using fully methylated positive controls, unmethylated negative controls, no-template controls, and serial dilutions of fully methylated DNA in each experiment. A common fluorescence threshold was applied for both *RNF152* and *ACTB*, and quantitative values for individual samples were obtained from the corresponding standard curves. Relative DNA methylation levels were calculated as the ratio of the quantitative *RNF152* value to the corresponding *ACTB* value and expressed as (*RNF152*/*ACTB*) x 100 as previously described (28). Methylation positivity was not defined using a percentage cut-off derived from the healthy control group. Samples showing reproducible methylation-specific *RNF152* amplification were classified as methylation-positive, whereas samples without detectable methylation-specific *RNF152* amplification were classified as methylation-negative. Accordingly, the absence of detectable *RNF152* promoter methylation in healthy oral mucosal tissues represented an observed result rather than a criterion used to establish methylation positivity.

### 2.6 Statistical analysis

Statistical analyses were performed using IBM SPSS Statistics version 31.0 (IBM Corp., Armonk, NY, USA). Continuous variables were summarized as median and range or mean ± standard deviation, as appropriate. Normality of continuous variables was assessed using the Shapiro-Wilk test. Because several continuous variables deviated from a normal distribution, non-parametric tests were used for the corresponding comparisons. Differences between independent groups were evaluated using the Mann-Whitney U test or the Kruskal-Wallis test, depending on the number of groups compared. Paired comparisons between lesion (OPML) or tumor (OSCC) tissues and their corresponding adjacent normal tissues were performed using the Wilcoxon signed-rank test. Associations between clinicopathological characteristics and categorical *RNF152* methylation or expression variables were assessed using the chi-square test or Fisher’s exact test, as appropriate. To account for multiple testing, Holm correction was applied to nominally significant *post-hoc* pairwise comparisons (uncorrected p < 0.05) following Kruskal-Wallis analyses. Single hypothesis tests and omnibus tests were not subjected to multiplicity correction. Statistical significance for these *post-hoc* pairwise comparisons was determined based on Holm-adjusted p-values. Spearman’s rank correlation analysis was performed to assess the relationship between continuous *RNF152* promoter methylation levels and the corresponding gene expression parameters. Receiver operating characteristic (ROC) curve analyses were performed to evaluate the diagnostic performance of *RNF152* methylation and expression parameters. The area under the ROC curve (AUC), 95% confidence intervals, sensitivity, specificity, p-values and optimal cut-off values were determined where applicable. Univariate logistic regression analyses were performed to evaluate potential predictors of methylation-associated *RNF152* expression loss. Variables with p < 0.25 in univariate logistic regression analyses were considered candidates for multivariable modeling. Multivariable logistic regression was performed only when a sufficient number of candidate variables and outcome events were available to permit model estimation. Effect size measures were reported where appropriate. Odds ratios (ORs) with 95% confidence intervals were reported for logistic regression analyses, whereas AUC values with 95% confidence intervals were used to quantify discriminatory performance in ROC analyses. A two-sided p-value < 0.05 was considered statistically significant.

## 3 Results

### 3.1 Clinicopathological characteristics of the study population

A total of 75 individuals were enrolled in this study, including 30 patients with OSCC, 30 patients with OPML and 15 healthy controls. Tumor/lesion tissues, matched adjacent normal tissues, serum and saliva samples were collected from patients with OSCC and OPML, whereas normal oral mucosal tissues, serum and saliva samples were obtained from healthy controls. The clinicopathological characteristics of the OSCC and OPML cohorts are summarized in **Tables 2** and **3**, respectively. The OSCC cohort comprised 30 patients with a mean age of 58.6 years (range, 33–85 years), including 22 (73.3%) males and 8 (26.7%) females. The tongue was the most frequently affected anatomical site (33.3%, 10/30), followed by the buccal mucosa (23.3%, 7/30). According to the AJCC TNM classification, most patients presented with stage IV disease (53.3%, 16/30). Lymph node metastasis was observed in 46.7% (14/30) of patients, whereas distant metastasis was detected in 6.7% (2/30). Smoking and alcohol consumption were reported by 63.3% (19/30) and 6.7% (2/30) of patients, respectively.

**Table 2:** Clinicopathological characteristics of patients with OSCC and their associations with *RNF152* promoter methylation and gene expression.

Expression/methylation results		Total	Tumor methylation n (%)	p	Saliva methylation n (%)	p	Decreased expression n (%)	p	Increased expression n (%)	p	Methylation-based expression loss n (%)	p
Clinical parameters												
OSCC Patients n (%)		**30 (100)**	**11 (36.67)**		**6 (20)**		**10 (33.33)**		**12 (40)**		**4 (13.33)**	
Age (mean ± SD)		59.03 ± 13.32	59.36 ± 12.4		61.17 ± 13.11		62.8 ± 13.6		53.75 ± 13.54		66.75 ± 12.21	
Age	≤ 50 years	8 (30.00)	3 (27.27)	0.656	–	0.145	2 (20)	1.000	5 (41.67)	0.210	1 (25)	1.000
	> 50 years	22 (70.00)	8 (72.73)		6 (100)		8 (80)		7 (58.33)		3 (75)	
Gender	Female	8 (26.67)	2 (18.18)	0.524	1 (16.67)	0.851	2 (20)	0.682	4 (33.33)	0.678	2 (50)	0.284
	Male	22 (73.33)	9 (81.82)		5 (83.33)		8 (80)		8 (66.67)		2 (50)	
Anatomical Region	Inferior Alveolar Arch	4 (13.33)	3 (27.27)	0.276	1 (16.67)	0.310	3 (30)	0.641	1 (8.33)	0.196	2 (50)	0.693
	Floor of Mouth	4 (13.33)	2 (18.18)		1 (16.67)		1 (10)		1 (8.33)		–	
	Buccal Mucosa	7 (23.33)	3 (27.27)		2 (33.33)		1 (10)		1 (8.33)		1 (25)	
	Retromolar Trigone	3 (10.00)	–		–		1 (10)		1 (8.33)		–	
	Tongue	10 (33.33)	3 (27.27)		1 (16.67)		4 (40)		6 (50)		1 (25)	
	Hard Palate	2 (6.67)			1 (16.67)				2 (16.67)		–	
Overall Stage	I	3 (10.00)	1 (9.09)	0.789	2 (33.33)	0.231	–	***0.038***	2 (16.67)	0.668	–	1.000
	II	6 (20.00)	2 (18.18)		1 (16.67)		3 (30)		2 (16.67)		1 (25)	
	III	5 (16.67)	1 (9.09)		–		4 (40)		1 (8.33)		1 (25)	
	IV	16 (53.33)	6 (54.55)		3 (50)		3 (30)		7 (58.33)		2 (50)	
	Early Stage	9 (30.00)	4 (36.36)	0.504	3 (50)	0.275	3 (30)	1.000	4 (33.33)	1.000	1 (25)	1.000
	Advanced Stage	21 (70.00)	7 (63.64)		3 (50)		7 (70)		8 (66.67)		3 (75)	
Differentiation	High	4 (13.33)	3 (27.27)	0.216	2 (33.33)	0.278	–	0.258	2 (16.67)	0.139	–	1.000
	Moderate	24 (80.00)	8 (72.73)		3 (50)		10 (100)		8 (66.67)		4 (100)	
	Low	2 (6.67)	–		0 ()		–		2 (16.67)		–	
Lymph Node Metastasis	(-)	16 (53.33)	8 (72.73)	0.341	4 (66.67)	0.457	6 (60)	1.000	5 (41.67)	0.274	3 (75)	0.606
	**(+)**	14 (46.67)	3 (27.27)		2 (33.33)		4 (40)		7 (58.33)		1 (25)	
Smoking	**(-)**	11 (36.67)	4 (36.36)	0.672	–	0.053	4 (40)	1.000	6 (50)	0.266	3 (75)	0.126
	**(+)**	19 (63.33)	7 (63.64)		6 (100)		6 (60)		6 (50)		1 (25)	
Alcohol	**(-)**	28 (93.33)	9 (81.82)	0.113	6 (100)	0.083	10 (100)	0.540	11 (91.67)	1.000	4 (100)	1.000
	**(+)**	2 (6.67)	2 (18.18)		–		–		1 (8.33)		–	
**Recurrence**	**(-)**	23	9 (81.82)	0.812	5 (83.33)	0.401	7 (70)	0.657	11 (91.67)	0.193	3 (75)	1.000
	**(+)**	7	2 (18.18)		1 (16.67)		3 (30)		1 (8.33)		1 (25)	
**Treatment**	Surgery	7	3 (27.27)	0.480	2 (33.33)	0.835	2 (20)	0.526	3 (25)	0.059	–	0.714
	Surgery + RT	19	8 (72.73)		2 (33.33)		8 (80)		5 (41.67)		4 (100)	
	Surgery + CRT	3	–		1 (16.67)		–		3 (25)		–	

Bold values indicate statistical significance (p < 0.05).

**Table 3:** Clinical and pathological data of the premalignant group.

Expression/ methylation results		Total	Tumor methylation n (%)	p	Saliva methylation n (%)	p	Decreased expression n (%)	p	Increased expression n (%)	p	Meth-based Exp Loss n (%)	p
Clinical parameters												
OPML Patients n (%)		**30 (100)**	**21 (70)**		**11 (36.67)**		**9 (30)**		**11 (36.67)**		**9 (30)**	
Age (mean ± SD)	52.5 ± 14.68		52.90 ± 15.07		52.63 ± 14.88		54.67 ± 15.02		51.01 ± 15.42		49 ± 15.09	
Age	≤ 50 years	15 (50)	10 (47.62)	0.958	5 (45.45)	0.488	4 (44.44)	1.000	5 (45.45)	1.000	4 (44.44)	0.642
	> 50 years	15 (50)	11 (52.38)		6 (54.55)		5 (55.56)		6 (54.55)		5 (55.56)	
Gender	Female	16 (53.33)	11 (52.38)	0.475	7 (63.64)	0.545	4 (44.44)	0.694	4 (36.36)	0.257	4 (44.44)	0.157
	Male	14 (46.67)	10 (47.62)		4 (36.36)		5 (55.56)		7 (63.64)		5 (55.56)	
Clinicopathological Diagnosis	Lichen Planus (LP)	16 (53.33)	13 (61.90)	0.056	4 (36.36)	0.526	4 (44.44)	0.872	6 (54.55)	1.000	4 (44.44)	0.670
	Oral Leukoplakia (OL)	8 (26.67)	4 (19.05)		4 (36.36)		3 (33.33)		3 (27.27)		3 (33.33)	
	Oral Lichenoid Dysplasia (OLD)	6 (20)	4 (19.05)		3 (27.27)		2 (22.22)		2 (18.18)		2 (22.22)	
Smoking	**(-)**	13 (43.33)	9 (38.10)	0.595	7 (63.64)	***0.023***	4 (44.44)	1.000	4 (36.36)	0.708	4 (44.44)	0.355
	**(+)**	17 (56.67)	13 (61.90)		4 (36.36)		5 (55.56)		7 (63.64)		5 (55.56)	
Alcohol	**(-)**	24 (80)	17 (80.95)	0.770	9 (81.82)	0.971	7 (77.78)	1.000	9 (81.82)	1.000	7 (77.78)	1.000
	**(+)**	6 (20)	4 (19.05)		2 (18.18)		2 (22.22)		2 (18.18)		2 (22.22)	
Family History	**(-)**	21 (75)	14 (73.69)	0.727	6 (60)	0.466	6 (75)	1.000	9 (90)	0.215	7 (77.78)	1.000
	**(+)**	7 (25)	5 (26.31)		4 (40)		2 (25)		1 (10)		2 (22.22)	

Bold values indicate statistical significance (p < 0.05).

The OPML cohort consisted of 30 patients with a mean age of 52.0 years (range, 27–83 years), including 14 (46.7%) males and 16 (53.3%) females. Oral lichen planus (OLP) was the most common diagnosis (53.3%, 16/30), followed by oral leukoplakia (OL) (26.7%, 8/30) and oral lichenoid dysplasia (OLD) (20.0%, 6/30). Within the OL subgroup, 62.5% (5/8) of patients were female and 37.5% (3/8) were male, with an age range of 31–56 years. Smoking and alcohol consumption were reported by 50.0% (4/8) and 25.0% (2/8) of patients, respectively. The OLD subgroup consisted predominantly of females (83.3%, 5/6) with one male patient (16.7%, 1/6), and an age range of 33–83 years. Smoking and alcohol consumption were reported by 50.0% (3/6) and 33.3% (2/6) of patients, respectively. In the OLP subgroup, females and males were equally represented (50.0%, 8/16 each), with ages ranging from 27 to 77 years. Smoking and alcohol consumption were reported by 62.5% (10/16) and 12.5% (2/16) of patients respectively. Overall, smoking and alcohol consumption were reported by 56.7% (17/30) and 20.0% (6/30) of OPML patients, respectively.

The healthy control group consisted of 15 individuals with a mean age of 54.7 years (range, 42–75 years), including 8 (53.3%) males and 7 (46.7%) females. Smoking and alcohol consumption were each reported by 26.7% (4/15) of individuals.

### 3.2 Expression of *RNF152* gene in OSCC and OPML

*RNF152* gene expression was evaluated in paired tumor or lesion and matched adjacent normal tissue samples obtained from patients with OSCC and OPML, using QRT-PCR. Relative expression levels were calculated using the 2^-ΔΔCt^ method. Samples with fold-change values ≥2 were classified as exhibiting increased *RNF152* expression, whereas samples with fold-change values ≤0.5 were classified as exhibiting decreased expression. For visualization purposes, downregulated samples are presented as negative reciprocal fold-change values in **Figure 1**. Decreased *RNF152* expression was detected in 33.3% (10/30) of OSCC cases and 30.0% (9/30) of OPML cases. Conversely, increased *RNF152* expression was observed in 40.0% (12/30) of OSCC patients and 36.7% (11/30) of OPML patients. The remaining samples showed no marked alteration in *RNF152* expression, indicating substantial inter-individual heterogeneity in both disease groups (**Figure 1**).

**Figure 1:**
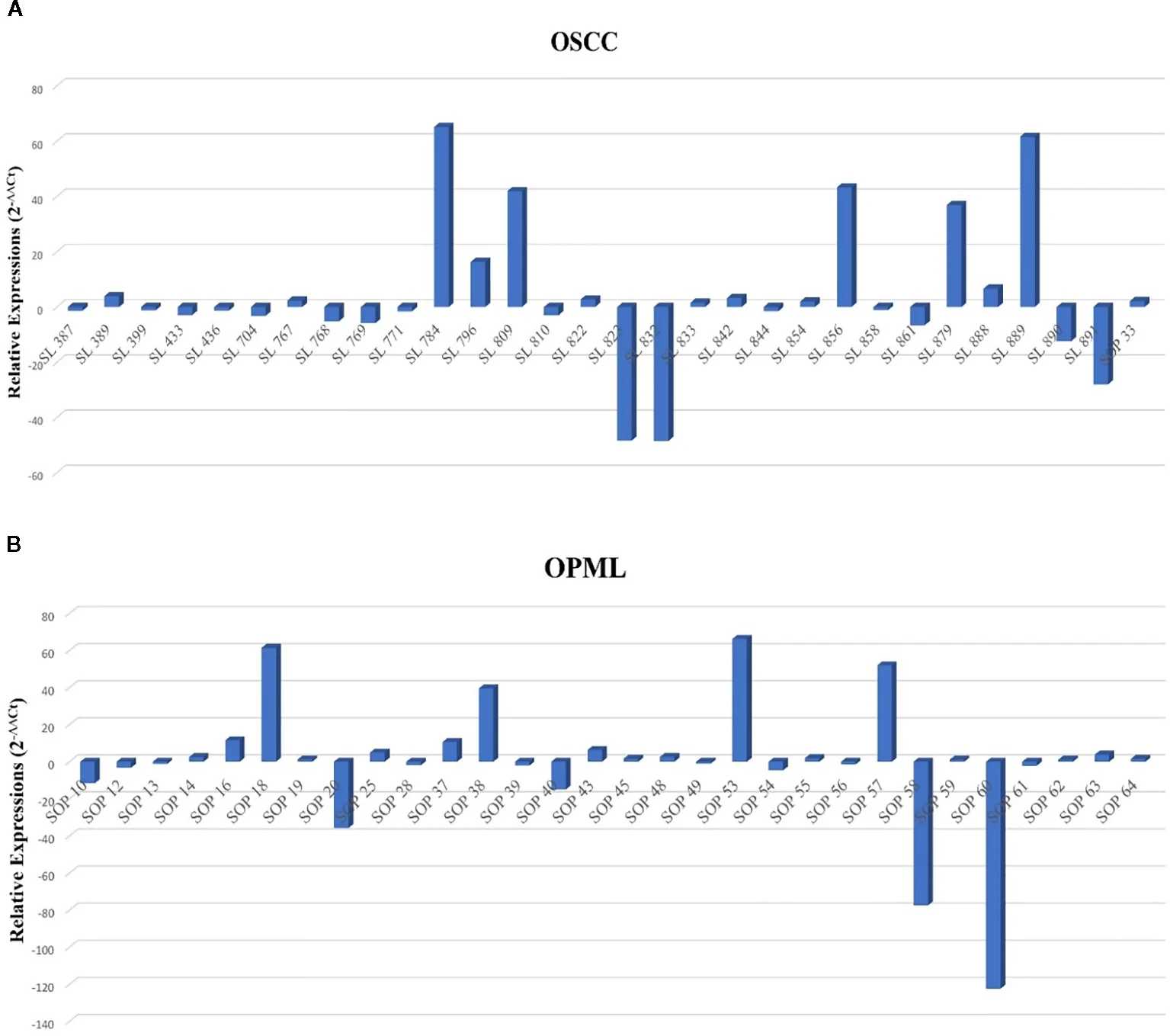
Relative *RNF152* expression profiles in **(a)** OSCC tumor tissues and **(b)** OPML lesion tissues compared with their matched adjacent normal tissues. (SOP, sample identification number).

When compared with healthy oral mucosa, *RNF152* expression was approximately 2.150-fold lower in OSCC tumor tissues and 2.653-fold lower in matched adjacent normal tissues. At the group level, tumor tissues exhibited approximately 1.233-fold higher *RNF152* expression than their matched adjacent normal tissues. Similarly, *RNF152* expression was approximately 1.920-fold lower in OPML lesions and 2.370-fold lower in matched adjacent normal tissues than in healthy oral mucosa. OPML lesion tissues exhibited approximately 1.234-fold higher *RNF152* expression than their corresponding matched adjacent normal tissues.

To evaluate changes in *RNF152* expression, paired comparisons were performed between tumor/lesion tissues and their matched adjacent normal tissues within each cohort using the Wilcoxon signed-rank test. No significant differences in *RNF152* expression levels were observed between OSCC tumor tissues and matched adjacent normal tissues (p = 0.727) or between OPML lesion tissues and matched adjacent normal tissues (p = 0.453).

Receiver operating characteristic (ROC) analyses were performed to evaluate the diagnostic performance of tissue *RNF152* expression (**Figure 2**). In the comparison between OSCC and OPML, tumor/lesion tissue ΔCt values demonstrated poor discriminatory performance, with an AUC of 0.552 (95% CI: 0.405-0.700, p = 0.487). The optimal cut-off value was 3.83, yielding a sensitivity of 63.3% and a specificity of 56.7% (Youden index = 0.20) (**Figure 2a**).

**Figure 2:**
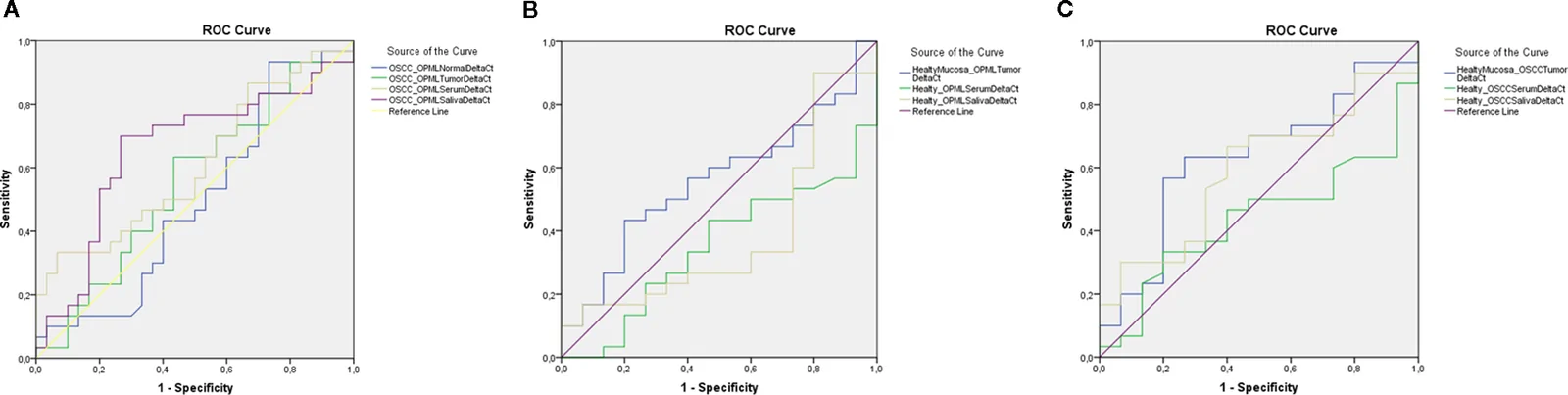
ROC curve analyses of *RNF152* expression. **(a)** Comparison between OSCC and OPML patients. **(b)** Comparison between OPML patients and healthy controls. **(c)** Comparison between OSCC patients and healthy controls. ROC curves were generated using ΔCt values from matched adjacent normal tissue, tumor/lesion tissue and liquid biopsy samples (serum and saliva), as appropriate for each comparison.

For distinguishing OPML patients from healthy controls, *RNF152* expression in matched adjacent normal tissues yielded an AUC of 0.649 (95% CI: 0.485-0.813, p = 0.107),with an optimal cut-off value of 3.27, sensitivity of 70.0% and specificity of 66.7% (Youden index = 0.367). Lesion tissue expression showed lower discriminatory performance (AUC = 0.560, 95% CI: 0.388-0.732, p = 0.516) with an optimal cut-off value of 3.72, sensitivity of 46.7% and a specificity of 73.3% (Youden index = 0.200). Thus, neither tissue parameter significantly discriminated OPML patients from healthy controls (**Figure 2b**).

For distinguishing OSCC patients from healthy controls, *RNF152* expression in matched adjacent normal tissues showed the highest diagnostic performance and was the only significant tissue expression parameter, with an AUC of 0.687 (95% CI: 0.513-0.860, p = 0.043), an optimal cut-off value of 3.08, sensitivity of 80.0% and specificity of 60.0% (Youden index = 0.400). Tumor tissue expression showed lower and non-significant discriminatory performance (AUC = 0.633, 95% CI: 0.462-0.805, p = 0.149), with an optimal cut-off value of 3.73, sensitivity of 63.3% and specificity of 73.3% (Youden index = 0.367) (**Figure 2c**).

### 3.3 Evaluation of *RNF152* expression in liquid biopsy samples

Relative *RNF152* expression levels were evaluated in serum and saliva samples from patients with OSCC and OPML and compared with the corresponding body-fluid samples from healthy controls. In the OSCC group, serum *RNF152* expression was modestly increased by 1.090-fold, whereas salivary expression was approximately 1.380-fold lower than in healthy controls. In the OPML group, *RNF152* expression was increased by 1.978-fold in serum and 1.310-fold in saliva relative to healthy controls.

ROC analyses were performed to evaluate the diagnostic performance of serum and salivary *RNF152* ΔCt values (**Figure 2**). For distinguishing OSCC from OPML, serum ΔCt values showed limited discriminatory performance (AUC = 0.611, 95% CI: 0.468-0.755, p = 0.139), with an optimal cut-off value of 1.31, sensitivity of 50.0% and specificity of 60.0% (Youden index = 0.100). In contrast, salivary ΔCt values significantly discriminated OSCC from OPML (AUC = 0.657, 95% CI: 0.512-0.801, p = 0.037), with an optimal cut-off value of 2.49, sensitivity of 70.0% and specificity of 73.3% (Youden index = 0.433) (**Figure 2a**).

For distinguishing OPML from healthy controls, neither serum nor salivary ΔCt values demonstrated significant discriminatory performance. Serum ΔCt values yielded an AUC of 0.350 (95% CI: 0.188-0.512, p = 0.104). Because the ROC curve was below the reference line and the Youden index did not exceed zero, no meaningful cut-off value was identified. Salivary ΔCt values similarly showed poor discriminatory performance (AUC = 0.387, 95% CI: 0.206-0.567, p = 0.219), with an optimal cut-off value of 4.25, sensitivity of 16.7% and specificity of 93.3% (Youden index = 0.100) (**Figure 2b**).

For distinguishing OSCC from healthy controls, serum ΔCt values showed poor discriminatory performance (AUC = 0.440, 95% CI: 0.270-0.610, p = 0.516), with an optimal cut-off value of 3.64, sensitivity of 33.3% and specificity of 80.0% (Youden index = 0.133). Salivary ΔCt values also showed limited and non-significant discriminatory performance (AUC = 0.594, 95% CI: 0.424-0.765, p = 0.306), with an optimal cut-off value of 2.66, sensitivity of 66.7% and specificity of 60.0% (Youden index = 0.267) (**Figure 2c**).

Consistent with the ROC findings, Mann-Whitney U analysis revealed no significant differences in serum *RNF152* ΔCt values between OSCC patients and healthy controls (p = 0.522) or between OPML patients and healthy controls (p = 0.086). Likewise, salivary *RNF152* ΔCt values did not differ significantly between OSCC patients and healthy controls (p = 0.248) or between OPML patients and healthy controls (p = 0.167).

### 3.4 Association of *RNF152* expression with clinicopathological characteristics

#### 3.4.1 OSCC

Clinicopathological associations of *RNF152* expression were evaluated in the OSCC cohort using fold-change values together with *RNF152* ΔCt values obtained from tumor tissues, matched adjacent normal tissues, serum and saliva samples. Most clinicopathological variables, including gender, smoking status, alcohol consumption, lymph node metastasis, histological differentiation and disease stage (early vs. advanced), were not significantly associated with *RNF152* expression parameters (all p > 0.05) (**Table 2**).

Age was significantly associated with *RNF152* expression in matched adjacent normal tissues (Mann-Whitney U test, exact p = 0.016). Patients aged ≤50 years exhibited higher median ΔCt values than those aged >50 years (5.04 vs. 3.58), indicating lower *RNF152* expression in the younger age group. No significant age-related differences were observed in tumor tissue, serum, salivary expression, or fold-change values (all p > 0.05) (**Table 2**).

Tumor recurrence was significantly associated with tumor tissue *RNF152* expression (Mann-Whitney U test, exact p = 0.037). Patients with recurrent disease exhibited higher median tumor tissue ΔCt values than those without recurrence (5.05 vs. 3.97), indicating lower *RNF152* expression in recurrent tumors. No significant differences were detected for serum, saliva or fold-change values (**Table 2**).

Although overall anatomical tumor location was not significantly associated with fold-change values or *RNF152* expression in tumor tissues or matched adjacent normal tissues, serum *RNF152* expression levels also did not differ significantly across anatomical subsites (Kruskal-Wallis test, p = 0.075). Exploratory pairwise analyses identified nominal differences in serum ΔCt values between tongue and retromolar trigone tumors (uncorrected p = 0.028) and between tongue and hard palate tumors (uncorrected p = 0.030). However, neither comparison remained statistically significant after Holm correction (both adjusted p = 0.420). An additional pairwise comparison between buccal mucosa and hard palate tumors was not statistically significant (uncorrected p = 0.056).

Treatment modality was not associated with fold-change values or *RNF152* expression levels in tumor tissues, matched adjacent normal tissues, or serum samples. However, salivary *RNF152* expression differed significantly among patients treated with surgery alone, surgery followed by radiotherapy, and surgery followed by concurrent chemoradiotherapy (Kruskal-Wallis test, p = 0.019). None of the nominally significant *post-hoc* pairwise comparisons among treatment groups remained statistically significant after Holm correction (adjusted p ≥ 0.075).

Tumor stage was not significantly associated with continuous *RNF152* expression parameters, including fold-change values (Kruskal-Wallis test, p = 0.098). Exploratory pairwise analysis identified a nominal difference in fold-change values between stage III and stage IV tumors (uncorrected p = 0.019). However, this difference did not remain statistically significant after Holm correction (adjusted p = 0.114).

When *RNF152* expression was evaluated categorically, the frequency of *RNF152* downregulation differed significantly across AJCC tumor stages (Stages I-IV) (Fisher-Freeman-Halton exact test, p = 0.038) with the highest frequency observed in stage III tumors (80.0%), followed by stage II (50.0%) and stage IV (18.8%), whereas no cases of *RNF152* downregulation were detected in stage I tumors (**Table 2**). In contrast, neither *RNF152* upregulation nor methylation-associated *RNF152* expression loss was significantly associated with the evaluated clinicopathological characteristics (all p > 0.05). Detailed associations between categorical *RNF152* expression patterns (downregulation, upregulation and methylation-associated expression loss) and clinicopathological characteristics are presented in **Table 2**.

#### 3.4.2 OPML

Clinicopathological associations of *RNF152* expression were evaluated in patients with OPML using fold-change values together with *RNF152* ΔCt values obtained from lesion tissues, matched adjacent normal tissues, serum and saliva samples. Alcohol consumption was significantly associated with serum *RNF152* expression (Mann-Whitney U test, exact p = 0.006). Alcohol consumers exhibited lower median serum ΔCt values than non-drinkers (-0.18 vs. 1.59), consistent with higher serum *RNF152* expression in the alcohol-consuming group. No significant associations were observed between alcohol consumption and tissue expression levels or fold change values (all p > 0.05).

Smoking status, gender, age, and family history were not significantly associated with any *RNF152* expression parameter (all p > 0.05). Likewise, no significant differences in *RNF152* expression were detected among the three OPML diagnostic subgroups, including expression in matched adjacent normal tissues (Kruskal-Wallis test, p = 0.096).

When *RNF152* expression was evaluated categorically, neither decreased nor increased *RNF152* expression was significantly associated with OPML diagnostic subgroup, smoking status, alcohol consumption, gender, age group, or family history (all Fisher’s exact test p > 0.05). Detailed associations between categorical *RNF152* expression patterns and clinicopathological characteristics are presented in **Table 3**.

### 3.5 Promoter methylation of *RNF152* gene in OSCC and OPML

*RNF152* promoter methylation was evaluated in paired tissue samples together with matched serum and saliva specimens obtained from patients with OSCC and OPML (**Figure 3**). In the OSCC cohort, promoter methylation was detected in 36.7% (11/30) of tumor tissues and 26.7% (8/30) of matched adjacent normal tissues. Promoter methylation was also identified in serum and saliva samples from 26.7% (8/30) and 30.0% (9/30) of patients, respectively. Concurrent methylation across all analyzed sample types (tumor tissue, matched adjacent normal tissue, serum and saliva) was observed in two patients.

**Figure 3:**
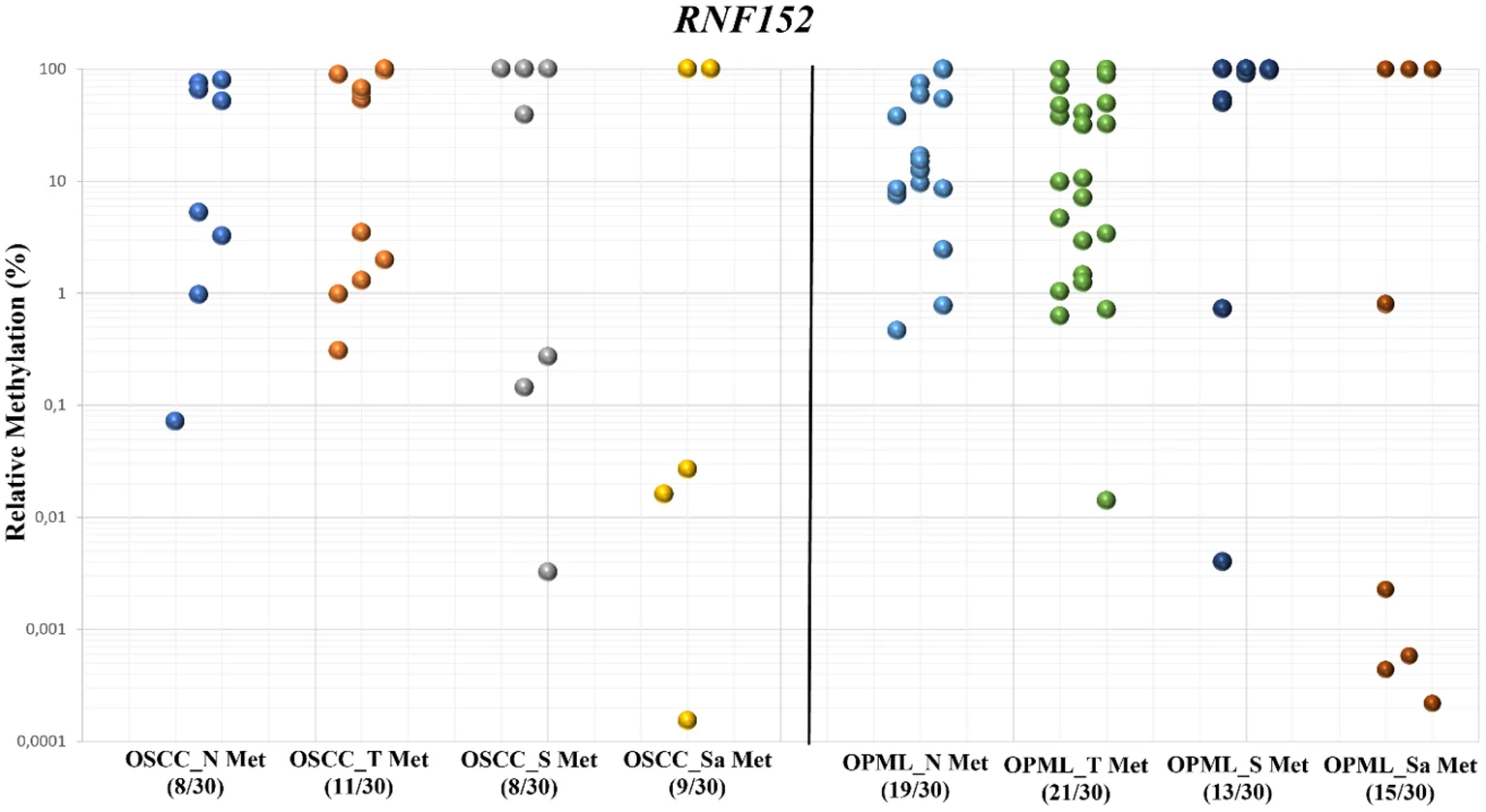
Relative *RNF152* promoter methylation levels in tissue and body fluid samples determined by QMSP. Methylation levels are expressed as the *RNF152/ACTB* ratio ×100. The y-axis is displayed on a logarithmic scale. (Met, methylation; N, matched adjacent normal tissue; T, tumor tissue; S, serum; Sa, saliva).

In the OPML cohort, *RNF152* promoter methylation was detected in 70.0% (21/30) of lesion tissues and 63.3% (19/30) of matched adjacent normal tissues. Promoter methylation was also identified in serum and saliva samples from 43.3% (13/30) and 50.0% (15/30) of patients, respectively, while concurrent promoter methylation across all analyzed sample types was observed in nine patients (**Figure 3**). The methylation frequency was significantly higher in OPML lesion tissues than in OSCC tumor tissues (70.0% vs 36.7%, p = 0.003).

*RNF152* promoter methylation was not detected in any of the oral mucosal tissues obtained from healthy controls. Consequently, methylation analysis was not extended to the corresponding serum and saliva samples.

Comparison of paired methylation levels using the Wilcoxon signed-rank test revealed no significant differences between OSCC tumor tissues and their matched adjacent normal tissues (p = 0.394), or between OPML lesion tissues and their corresponding matched adjacent normal tissues (p = 0.833).

ROC curve analysis was performed to evaluate the diagnostic performance of *RNF152* promoter methylation levels in distinguishing OPML from OSCC cases. Methylation levels in matched adjacent normal tissues significantly discriminated OPML from OSCC, with an AUC of 0.692 (95% CI: 0.555-0.829, p = 0.011). The optimal cut-off value was 0.27, yielding a sensitivity of 63.3% and a specificity of 76.7% (Youden index = 0.400). Similarly, lesion/tumor tissue methylation levels significantly differentiated OPML from OSCC cases (AUC = 0.663, 95% CI: 0.521-0.806, p = 0.030), with an optimal cut-off value of 0.47, sensitivity of 70.0% and specificity of 66.7% (Youden index = 0.367). In contrast, serum methylation levels demonstrated limited and non-significant discriminatory performance (AUC = 0.594, 95% CI: 0.449-0.739, p = 0.209), while salivary methylation levels were also non-significant (AUC = 0.626, 95% CI: 0.484-0.768, p = 0.093). The optimal cut-off values were 0.50 for serum and 100 for saliva, corresponding to sensitivities of 40.0% and 30.0% and specificities of 83.3% and 100%, respectively. Thus, tissue methylation levels significantly discriminated OPML from OSCC, whereas serum and salivary methylation levels did not (**Figure 4a**).

**Figure 4:**
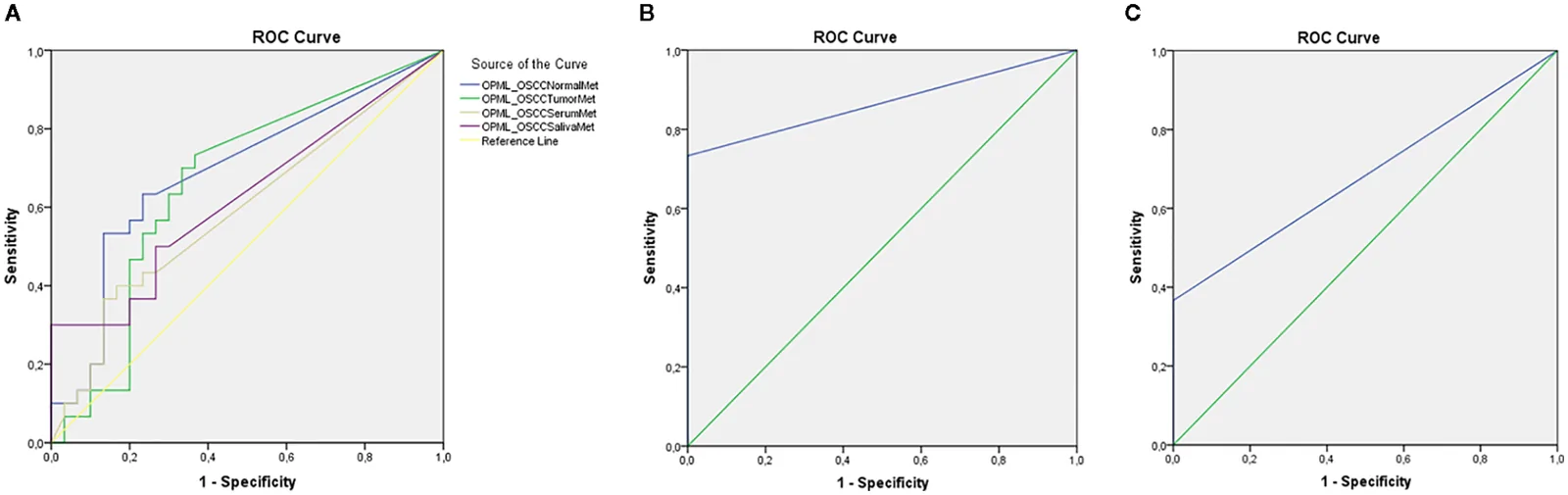
ROC curve analyses of *RNF152* promoter methylation. **(a)** Comparison between patients with OSCC and OPML using the corresponding tissue and liquid biopsy samples. **(b)** Comparison between OPML lesion tissues and healthy oral mucosa. **(c)** Comparison between OSCC tumor tissues and healthy oral mucosa.

ROC analysis was subsequently performed to evaluate tissue *RNF152* promoter methylation levels for distinguishing OPML and OSCC from healthy controls. Lesion tissue methylation showed good discriminatory performance for OPML versus healthy controls (AUC = 0.867, 95% CI: 0.762-0.971, p < 0.001), with an optimal cut-off value of 0.007, sensitivity of 73.3% and specificity of 100.0% (Youden index = 0.733) (**Figure 4b**). Tumor tissue methylation showed statistically significant but more modest discriminatory performance for OSCC versus healthy controls (AUC = 0.683, 95% CI: 0.530-0.836, p = 0.047), with an optimal cut-off value of 0.153, sensitivity of 36.7% and specificity of 100.0% (Youden index = 0.367) (**Figure 4c**). The observed AUC, sensitivity, and Youden index were higher for the OPML-versus-healthy comparison than for the OSCC-versus-healthy comparison.

### 3.6 Association of *RNF152* methylation with clinicopathological characteristics

Associations between *RNF152* methylation parameters and clinicopathological characteristics were investigated in patients with OSCC (**Table 2**). Smoking status was not significantly associated with *RNF152* methylation levels in matched adjacent normal tissue, tumor tissue, or serum samples (all p > 0.05), although salivary methylation demonstrated a borderline association with smoking (exact p = 0.053) (**Table 2**). Similarly, alcohol consumption was not significantly associated with tumor tissue or salivary methylation, whereas serum *RNF152* methylation was significantly associated with alcohol consumption (exact p = 0.021) (**Table 2**).

No significant associations were observed between *RNF152* methylation parameters and lymph node metastasis, recurrence, age, gender, anatomical tumor site, histopathological differentiation, or treatment modality (all p > 0.05) (**Table 2**). Likewise, tumor stage was not significantly associated with *RNF152* methylation parameters, either across AJCC stages I-IV (p = 0.094 for serum methylation) or when categorized as early versus advanced stage (exact p = 0.098 for serum methylation) (**Table 2**).

Associations between *RNF152* promoter methylation parameters and clinicopathological characteristics were also evaluated in patients with OPML (**Table 3**). Smoking status was significantly associated only with salivary *RNF152* methylation (Mann-Whitney U test, exact p = 0.023), whereas no significant associations were observed for lesion tissue, matched adjacent normal tissue or serum methylation parameters (all p > 0.05). Alcohol consumption, gender and family history were not significantly associated with any *RNF152* methylation parameter (all p > 0.05). Age was likewise not significantly associated with *RNF152* methylation parameters, including serum methylation (exact p = 0.068) (**Table 3**).

Overall, methylation-associated *RNF152* expression loss was identified in 30.0% (9/30) of patients with OPML and 13.3% (4/30) of patients with OSCC. Among methylation-positive cases, concomitant *RNF152* expression loss was observed in 4 of 11 OSCC cases (36.4%) and 9 of 21 OPML cases (42.9%). Thus, although promoter methylation and reduced expression were concordant in a subset of methylation-positive cases, methylation was not invariably accompanied by transcriptional downregulation.

In patients with OSCC, methylation-associated *RNF152* expression loss was not significantly associated with anatomical tumor location, tumor stage, disease stage, smoking status, alcohol consumption, gender, lymph node involvement, tumor differentiation, age, recurrence, or treatment modality (all p > 0.05). Similarly, in patients with OPML, methylation-associated *RNF152* expression loss was not significantly associated with diagnostic subgroup, smoking status, alcohol consumption, gender, age group, or family history (all p > 0.05). Univariate logistic regression analyses likewise did not identify statistically significant predictors of methylation-associated *RNF152* expression loss in either cohort.

Spearman’s rank correlation analysis was performed to evaluate the relationship between continuous *RNF152* promoter methylation levels and gene expression in the corresponding tissue samples. No significant correlation was observed between tumor tissue methylation levels and *RNF152* expression in the OSCC cohort (ρ = -0.018, p = 0.926) or between lesion tissue methylation levels and *RNF152* expression in the OPML cohort (ρ = 0.146, p = 0.441). Similarly, promoter methylation levels were not significantly correlated with tissue fold-change values in either OSCC (ρ = -0.042, p = 0.824) or OPML (ρ = -0.140, p = 0.460).

### 3.7 Analyses of *RNF152* promoter methylation and expression in OPML diagnostic subgroups

The distribution of *RNF152* promoter methylation and expression was further evaluated according to OPML subgroups. In the OLP subgroup, promoter methylation was detected in adjacent normal tissues, lesion tissues, serum and saliva samples in 62.5% (10/16), 81.25% (13/16), 25.0% (4/16), and 25.0% (4/16) of patients, respectively. All patients with promoter methylation in matched adjacent normal tissues also exhibited promoter methylation in the corresponding lesion tissues. Decreased *RNF152* expression was observed in 25.0% (4/16) of patients, whereas increased expression was detected in 37.5% (6/16). All patients with decreased *RNF152* expression also demonstrated promoter methylation in the corresponding lesion tissues (**Figure 5**).

**Figure 5:**
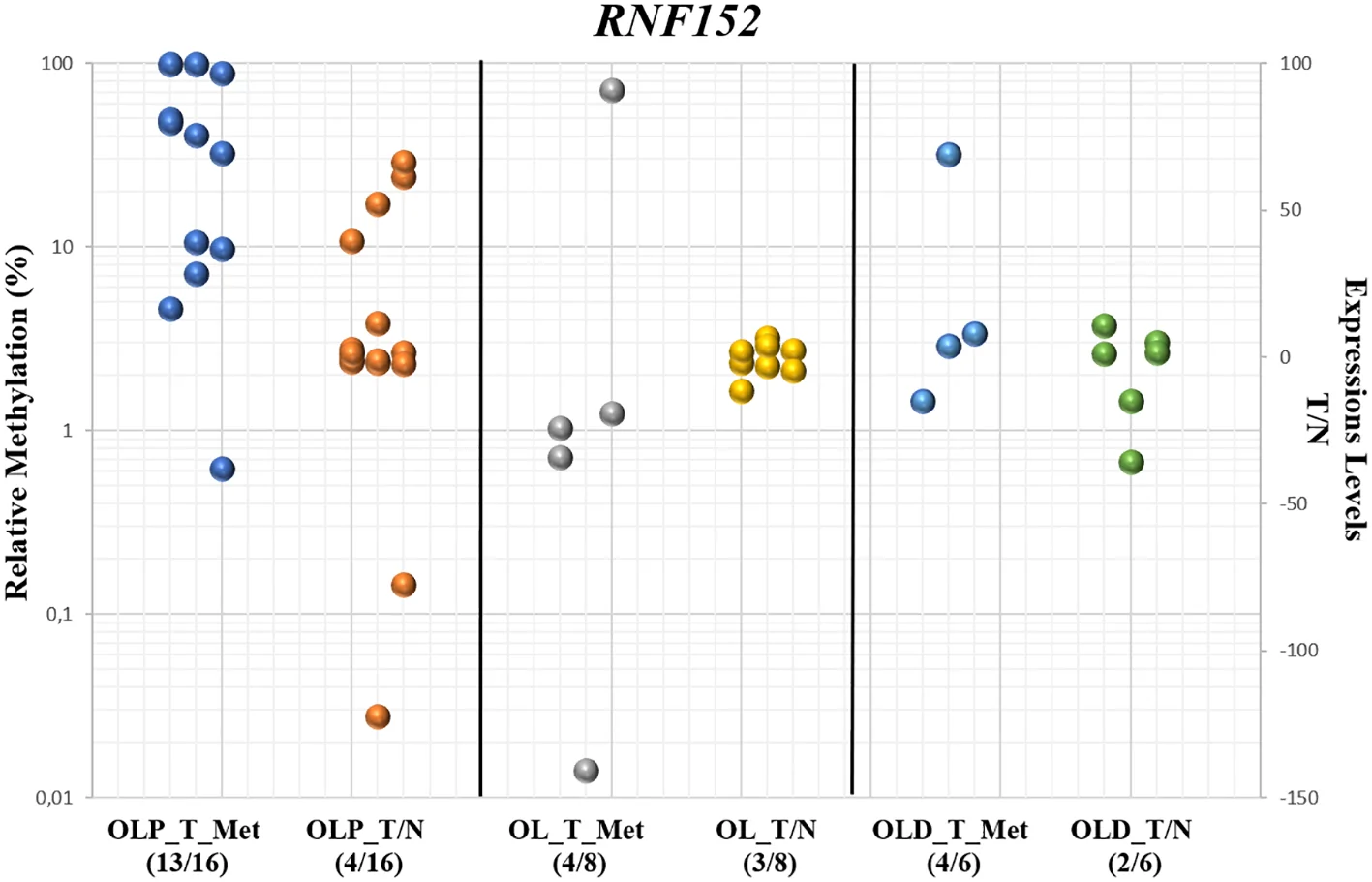
Distribution of *RNF152* promoter methylation and expression levels across the OPML diagnostic subgroups. Each dot represents an individual patient. The left y-axis indicates relative promoter methylation (%), whereas the right y-axis indicates *RNF152* expression (fold change). Numbers in parentheses indicate the number of promoter methylation-positive cases (T-Met) and the number of cases showing methylation-associated expression loss (T/N) in each subgroup. (OLP, oral lichen planus; OL, oral leukoplakia; OLD, oral lichenoid dysplasia).

In the OL subgroup, promoter methylation was detected in matched adjacent normal tissues, lesion tissues, serum and saliva samples in 62.5% (5/8), 50.0% (4/8), 50.0% (4/8) and 50.0% (4/8) of patients, respectively. All patients with promoter methylation in matched adjacent normal tissues also exhibited serum methylation, while 37.5% (3/8) of patients showed promoter methylation in all analyzed sample types. *RNF152* expression was decreased in 37.5% (3/8) and increased in 37.5% (3/8) of patients. All patients with decreased *RNF152* expression, also demonstrated promoter methylation in the corresponding lesion tissues (**Figure 5**).

In the OLD subgroup, promoter methylation was detected in matched adjacent normal tissues, lesion tissues, serum and saliva samples in 66.7% (4/6), 66.7% (4/6), 66.7% (4/6), and 50.0% (3/6) of patients, respectively. The patients with promoter methylation in matched adjacent normal tissues were the same individuals in whom serum methylation was detected, and 50.0% (3/6) of patients demonstrated promoter methylation in all analyzed sample types. Decreased *RNF152* expression was observed in 33.3% (2/6) of patients, whereas increased expression was detected in 33.3% (2/6). Both patients with decreased *RNF152* expression also exhibited promoter methylation in the corresponding lesion tissues (**Figure 5**).

Across all three OPML diagnostic subgroups, all nine patients with decreased *RNF152* expression also exhibited promoter methylation in the corresponding lesion tissue. However, because promoter methylation was detected in 21 OPML lesions overall, reduced expression was present in only a subset of methylation-positive cases (9/21, 42.9%) (**Figure 5**).

*RNF152* promoter methylation and expression patterns were compared among the three OPML diagnostic subgroups (OLP, OL and OLD). No statistically significant differences were observed in *RNF152* promoter methylation or expression parameters among the diagnostic subgroups (all p >0.05), including lesion tissue methylation (Kruskal-Wallis test, p = 0.056) and *RNF152* expression in matched adjacent normal tissues (p = 0.096) (**Table 3**).

Clinicopathological associations were subsequently evaluated within each OPML diagnostic subgroup. In the OL subgroup, *RNF152* methylation levels in matched adjacent normal tissues were significantly associated with gender (p = 0.036), while serum and salivary methylation levels were significantly associated with alcohol consumption (p = 0.036) and smoking status (p = 0.018), respectively. No significant associations were observed between *RNF152* expression parameters and the evaluated clinicopathological variables, including age and lesion tissue expression (exact p = 0.071). In the OLP subgroup, smoking status was significantly associated with salivary *RNF152* methylation levels (p = 0.036), whereas alcohol consumption was significantly associated with *RNF152* expression levels in both serum and saliva samples (p = 0.033 for both comparisons). In the OLD subgroup, no significant associations were identified between *RNF152* expression or methylation parameters and any evaluated clinicopathological characteristic (all p > 0.05). Given the small subgroup sizes and exploratory nature of these analyses, these nominal associations should be interpreted cautiously.

## 4 Discussion

Head and neck squamous cell carcinoma (HNSCC), with OSCC as its predominant subtype, remains a major global health burden despite substantial advances in molecular oncology. One of the principal challenges in oral cancer management is the lack of reliable biomarkers capable of detecting disease at an early stage and improving molecular risk stratification of premalignant lesions. Among the molecular alterations involved in oral carcinogenesis, aberrant DNA methylation represents one of the earliest and best-characterized epigenetic events. Owing to its stability, tissue specificity, detectability in minimally invasive samples, and potential reversibility, promoter methylation has emerged as an attractive source of molecular biomarkers (23, 29). Recent comprehensive reviews further emphasize the central role of epigenetic dysregulation, including aberrant DNA methylation, in OSCC pathogenesis and biomarker development (30, 31). A broad spectrum of locus-specific methylation alterations has been investigated in HNSCC and OSCC. Previous studies from our group and others have identified epigenetic alterations involving DNA mismatch-repair genes (32), *p16INK4a* (33), *KIF1A* and *EDNRB* (34, 35), *GNG7* (21), *NOL4* (36), *NID2* and *HOXA9* (22), as well as *DCC* (37). These studies demonstrated that promoter methylation occurs across multiple molecular pathways and may exhibit substantial intertumoral heterogeneity. Importantly, methylation-based detection has also been extended beyond tissue: *KIF1A* and *EDNRB* methylation was detectable in salivary rinses from patients with HNSCC (34), and salivary *EDNRB* methylation was subsequently associated with oral premalignancy and malignancy (35). More recent studies have continued to expand the methylation biomarker landscape. Sorroche et al. reported increased *HOXA1* 3′UTR methylation in OSCC tissues and associations with clinicopathological characteristics (38), whereas Lin et al. demonstrated frequent *SOX1* and *PAX1* methylation and improved diagnostic sensitivity when these markers were combined (39). Collectively, these findings highlight both the diversity of methylation alterations in oral carcinogenesis and the potential value of complementary multimarker strategies.

In the present study, we comprehensively evaluated *RNF152* promoter methylation together with its gene expression profile in patients with OPML and OSCC using paired tissue specimens and matched saliva and serum samples. To the best of our knowledge, this is the first study to investigate *RNF152* methylation and expression simultaneously across premalignant and malignant oral lesions while also evaluating its detectability and diagnostic performance in matched liquid-biopsy specimens. A particularly notable finding was the significantly higher frequency of *RNF152* promoter methylation in OPML (70.0%) than in OSCC (36.7%), whereas no promoter methylation was detected in healthy oral mucosa. This distribution suggests that *RNF152* methylation is associated with diseased oral epithelium and may arise relatively early during oral carcinogenesis. However, although oral carcinogenesis involves progressive molecular evolution, individual epigenetic alterations need not accumulate monotonically from premalignancy to invasive carcinoma and may be retained, remodeled or differentially selected during clonal evolution. Importantly, the present study is cross-sectional and compares independent OPML and OSCC cohorts rather than longitudinally following individual lesions through malignant transformation. Therefore, the lower methylation frequency in OSCC should not be interpreted as evidence of methylation loss during progression. Instead, the observed pattern may reflect biological heterogeneity, clonal selection, or epigenetic remodeling in established carcinomas. Longitudinal studies are required to determine the temporal dynamics of *RNF152* methylation and whether its presence in OPML is associated with subsequent malignant transformation.

Our findings extend the genome-wide observations of Poage et al. (15), in whose HNSCC methylation-array dataset an *RNF152*-associated CpG locus (cg19423014) was identified among loci associated with LINE-1 methylation. However, *RNF152* was not specifically investigated or independently validated as a promoter methylation target in that study, and its methylation prevalence and clinical significance in OSCC or OPML remained unknown. The present study extends these observations by independently evaluating *RNF152* promoter methylation in clinically characterized OPML and OSCC cohorts and by integrating methylation status with gene expression and matched liquid-biopsy analyses. Importantly, the detection of *RNF152* methylation in OPML demonstrates that this alteration is not restricted to established invasive carcinoma, although its temporal relationship with malignant progression remains to be determined.

The biological characteristics of *RNF152* further support its potential relevance to oral carcinogenesis. *RNF152* encodes a lysosome-associated RING-finger E3 ubiquitin ligase involved in ubiquitin-mediated regulation of intracellular signaling. Dysregulation of ubiquitination contributes to multiple cancer-related processes, including proliferation, apoptosis, metabolic regulation, and intracellular signaling (40). Of particular relevance, *RNF152* negatively regulates mTORC1 signaling through ubiquitination of the small GTPases Rheb and RagA (17), thereby restricting cellular growth and promoting autophagy under nutrient-deprived conditions. Experimental loss of *RNF152* function has accordingly been associated with mTORC1 hyperactivation and impaired amino acid starvation-induced autophagy (41). These observations provide a biological rationale for investigating *RNF152* dysregulation in oral carcinogenesis, although the functional consequences of *RNF152* promoter methylation in oral lesions remain to be established.

Previous experimental studies further support the tumor-suppressive function of *RNF152* in epithelial malignancies. Wan et al. (2021) demonstrated that restoration of *RNF152* expression suppresses hepatocellular carcinoma progression by inhibiting mTORC1 signaling, inducing autophagy and apoptosis, and downregulating *CXCL6* expression through TSPAN12-mediated signaling (42). Similarly, Cui et al. (2018) reported that *RNF152* suppresses colorectal cancer cell proliferation, promotes apoptotic cell death, and is associated with favorable clinical outcomes (18). Collectively, these studies support a tumor-suppressive role for *RNF152* in several epithelial malignancies. In the present study, however, reduced *RNF152* expression was observed in only a subset of OSCC and OPML cases, and promoter methylation was not uniformly accompanied by transcriptional downregulation. This divergence suggests that *RNF152* regulation in oral carcinogenesis may be more heterogeneous than that observed in these experimental models and may involve multiple regulatory mechanisms beyond promoter methylation.

Interestingly, *RNF152* expression exhibited considerable heterogeneity within both disease groups. Increased expression was observed in 40.0% of OSCC and 36.7% of OPML cases, whereas reduced expression was detected in 33.3% and 30.0%, respectively. Divergent *RNF152* expression patterns have also been reported across other malignancies. Increased *RNF152* expression has been described in lung adenocarcinoma (19), whereas reduced expression and tumor-suppressive activity have been demonstrated in hepatocellular carcinoma (42) and colorectal cancer (18). These apparently contrasting observations indicate that *RNF152* expression and biological activity may vary according to tumor type and molecular context. In the present cohorts, the coexistence of *RNF152* upregulation, downregulation, and unchanged expression further suggests that its transcriptional regulation is heterogeneous and cannot be explained by promoter methylation alone. Potential contributors include differences in CpG methylation density and distribution, chromatin accessibility and histone modifications, transcription factor activity, post-transcriptional regulation by non-coding RNAs, tumor cellular composition, and intertumoral molecular heterogeneity. Clonal selection during tumor evolution may further generate distinct *RNF152* regulatory states among individual lesions. These mechanisms provide plausible explanations for the heterogeneous expression patterns observed in both OPML and OSCC and warrant further mechanistic investigation.

Although no statistically significant overall difference in *RNF152* expression was observed between paired lesion or tumor tissues and their corresponding adjacent normal tissues, methylation-associated *RNF152* expression loss was observed in 13.3% of OSCC and 30.0% of OPML cases. Among methylation-positive cases, concomitant *RNF152* expression loss was observed in only 36.4% of OSCC and 42.9% of OPML patients. Notably, all nine OPML cases showing reduced *RNF152* expression were methylation-positive, however, reduced expression was observed in only 9 of the 21 methylation-positive OPML cases. Moreover, analyses using continuous measurements did not demonstrate a significant correlation between *RNF152* promoter methylation levels and tissue expression in either cohort. Collectively, these findings suggest that promoter methylation may contribute to *RNF152* transcriptional regulation in a subset of cases but is not sufficient to account for the heterogeneous expression patterns observed across the cohorts. Importantly, the present data demonstrate an association and do not establish a direct causal relationship between *RNF152* promoter methylation and transcriptional repression.

Incomplete methylation-expression concordance is not unique to *RNF152*. In our previous study of p16INK4a in head and neck cancer, promoter methylation and expression loss were concordant in 48% of patients, while discordant methylation-expression patterns were also observed, indicating the contribution of additional regulatory mechanisms (33). Recent studies further illustrate the gene-specific nature of methylation-expression relationships in OSCC. *ZNF880* hypermethylation was associated with reduced expression in an independent validation cohort (43), whereas promoter hypermethylation of *GABRB2* was accompanied by expression loss in only a subset of methylated tumors (24). Together, these observations emphasize that the transcriptional consequences of promoter methylation vary among genes and tumors and should not be inferred solely from methylation status. Functional validation using demethylating agents such as 5-aza-2’-deoxycytidine, together with locus-specific methylation analyses by bisulfite sequencing or pyrosequencing, will therefore be required to determine the direct contribution of promoter methylation to *RNF152* transcriptional regulation.

Beyond its biological implications, tissue *RNF152* promoter methylation showed good discriminatory performance for OPML versus healthy oral mucosa (AUC = 0.867; sensitivity, 73.3%), whereas its performance for OSCC was statistically significant but more modest (AUC = 0.683; sensitivity, 36.7%), despite 100% specificity. These findings indicate that *RNF152* methylation lacks sufficient sensitivity for use as a standalone diagnostic biomarker, particularly for OSCC. Its potential clinical value may instead lie in incorporation into multimarker epigenetic panels containing complementary markers, an approach increasingly supported by methylation-based studies in oral cancer (39, 44).

An additional exploratory finding was that *RNF152* expression in histologically normal tissue adjacent to OSCC showed the highest discriminatory performance among the evaluated expression parameters and was the only expression parameter that significantly distinguished OSCC from healthy controls. This observation may reflect field cancerization, whereby molecular alterations extend into histologically normal epithelium beyond the visible tumor margins. However, because this was not a predefined primary endpoint, its biological and diagnostic significance requires independent validation.

The evaluation of matched saliva and serum samples further assessed the detectability of *RNF152* promoter methylation in liquid-biopsy specimens. Although methylation was detectable in both sample types, detection alone did not translate into adequate diagnostic performance, which was consistently inferior to that observed in tissue. A recent systematic review by Rapado-González et al. demonstrated substantial variability in the diagnostic performance of DNA methylation markers across non- and minimally invasive specimens, including saliva/oral rinse and blood, highlighting the influence of both the individual methylation marker and specimen type on diagnostic accuracy (45). Several biological and pre-analytical factors may contribute to the limited performance observed in the present study. In serum, tumor-derived circulating cell-free DNA may constitute only a small fraction of total cell-free DNA and can be diluted by DNA released from non-neoplastic cells, while fragmentation and degradation may further reduce the amount of amplifiable methylated target DNA. Saliva provides more direct access to oral epithelial material but contains DNA from multiple cellular sources and remains biologically heterogeneous. Thus, greater local detectability does not necessarily translate into adequate diagnostic discrimination. Accordingly, the present findings do not support *RNF152* methylation as a standalone liquid-biopsy biomarker but justify its further evaluation as part of multimarker epigenetic panels.

Clinicopathological analyses identified several associations involving *RNF152* methylation or expression; however, these analyses were exploratory, and several nominal *post-hoc* pairwise differences did not remain statistically significant after Holm correction for multiple testing. These findings should therefore be regarded as hypothesis-generating rather than confirmatory.

The present study intentionally evaluated OPML as a clinically relevant group of oral potentially malignant disorders rather than as isolated disease entities. Although OL, OLP, and OLD differ in their histopathological characteristics and malignant transformation risks, they all represent lesions with recognized malignant potential. Accordingly, primary analyses were performed in the overall OPML cohort to investigate *RNF152* molecular alterations across oral potentially malignant disorders, while separate subtype analyses were conducted to address their biological heterogeneity. Given the limited numbers within individual subgroups, these subtype-specific analyses should be considered exploratory and require confirmation in larger cohorts specifically designed for subtype-level evaluation.

Nevertheless, several limitations should be acknowledged. First, although sensitivity analysis indicated that the overall cohort was adequate to detect an overall effect of the magnitude specified in the statistical analysis, the small sizes of the individual OPML diagnostic subgroups limited the statistical power of subtype-specific analyses. Moreover, the small number of methylation-associated *RNF152* expression loss events, particularly in the OSCC cohort, limited the statistical power and stability of the logistic regression estimates, which should therefore be considered exploratory. Second, the cross-sectional design precluded assessment of longitudinal outcomes, including malignant transformation, and the absence of an independent external validation cohort limits the generalizability of the findings. Larger prospective, multicenter studies are therefore required to confirm the reproducibility and diagnostic performance of *RNF152*. Third, although our findings demonstrate an association between *RNF152* promoter methylation and reduced expression in a subset of cases, they do not establish causality. Functional validation using demethylating agents, together with locus-specific methylation analyses such as bisulfite sequencing or pyrosequencing, will be required to determine the direct contribution of promoter methylation to *RNF152* transcriptional regulation.

Despite these limitations, the study has several methodological strengths. To the best of our knowledge, this is the first study to evaluate *RNF152* promoter methylation together with gene expression across both oral premalignant and malignant lesions while simultaneously investigating matched saliva and serum specimens. The inclusion of paired lesion/tumor and adjacent tissues together with matched liquid-biopsy specimens from the same individuals reduced inter-individual variability and enabled direct comparison of *RNF152* alterations across clinically relevant specimen types.

In conclusion, *RNF152* promoter methylation was detected in oral premalignant and malignant lesions but was absent in healthy oral mucosa, supporting its association with diseased oral epithelium. Its diagnostic performance was stronger for OPML than for OSCC, while the limited sensitivity in OSCC and modest performance in liquid-biopsy specimens do not support its use as a standalone diagnostic biomarker. Rather, *RNF152* may represent a candidate component of multimarker epigenetic panels for molecular detection of oral lesions. Larger prospective and independent studies are required to validate its diagnostic contribution and determine whether its combination with complementary methylation markers improves clinical performance.

## Data Availability

The raw data supporting the conclusions of this article will be made available by the authors, without undue reservation. The publicly available microarray dataset from Poage et al. (2011), which informed the selection of RNF152 for targeted validation, is available in the NCBI Gene Expression Omnibus (GEO) under accession number GSE25093.
